# Supplementary material for: Genomic alterations and possible druggable mutations in carcinoma of unknown primary (CUP)
Source: Sci Rep. 2021 Jul 23;11:15112. doi: 10.1038/s41598-021-94678-4 (PMC8302572; doi:10.1038/s41598-021-94678-4)
Supplement: Supplementary file 1 — Supplementary Information 1. [file 41598_2021_94678_MOESM1_ESM.docx]

**Genomic Alterations and Possible Druggable Mutations in Carcinoma of Unknown Primary (CUP)**

Hamidreza Aboulkheyr Es^1, 2^, Hamid Mahdizadeh^1,3^, Amir Abbas Hedayati Asl^3^, Mehdi Totonchi^1,3, 4*^

1. Department of Genetics, Reproductive Biomedicine Research Center, Royan Institute for Reproductive Biomedicine, ACECR, Tehran, Iran.
2. School of Biomedical Engineering, University of Technology Sydney, Sydney, Australia.
3. Department of Stem Cells and Developmental Biology, Cell Science Research Center, Royan Institute for Stem Cell Biology and Technology, ACECR, Tehran, Iran.
4. School of Biological Sciences, Institute for Research in Fundamental Sciences (IPM), Tehran, Iran.

**This article contains five supplementary excel files:**

- **Supplementary Table-1: Identified significant mutated genes in CUP samples.**
- **Supplementary Table-2: The result of pathway enrichment analysis of SMGs from MSigDB.**
- **Supplementary Table-3: Co-occurrence and mutual exclusivity across identified SMG in CUP samples.**
- **Supplementary Table-4: Copy number alterations, amplifications, and deletion of CUP-SMG.**
- **Supplementary Table-5: The results gene mutation-drug association analysis from PanDrug platforms.**
